# Historical Control Analysis Demonstrates Greater Long‐Term Reduction in Plasma Globotriaosylceramide (Gb3) by Venglustat Compared With Placebo or Agalsidase Beta in Male Patients With Classic Fabry Disease

**DOI:** 10.1002/mgg3.70152

**Published:** 2025-11-17

**Authors:** Dominique P. Germain, Pronabesh DasMahapatra, Shiguang Liu, Patrick Deegan, Alberto Ortiz, Vijay Modur, William R. Wilcox

**Affiliations:** ^1^ Division of Medical Genetics University of Versailles Montigny France; ^2^ French Referral Center for Fabry Disease and Lysosomal Diseases, MetabERN AP‐HP Paris‐Saclay University Garches France; ^3^ Sanofi Cambridge Massachusetts USA; ^4^ Lysosomal Disorders Unit, Addenbrooke's Hospital Cambridge University Hospitals NHS Foundation Trust Cambridge UK; ^5^ Research Institute‐Fundacion Jimenez Diaz Autónoma University (IIS‐FJD UAM) Madrid Spain; ^6^ Division of Medical Genetics, Department of Human Genetics Emory University School of Medicine Atlanta Georgia USA

**Keywords:** Fabry disease, glucosylceramide synthase, glycosphingolipid synthesis, lysosomal disorder, substrate reduction therapy, venglustat

## Abstract

**Purpose:**

To evaluate the disease biomarker response of venglustat in patients with Fabry disease (FD), utilizing data from a single‐arm phase 2 study of venglustat and a placebo‐controlled phase 3 study of agalsidase beta through historical control and case‐matched analyses.

**Methods:**

Eleven venglustat‐treated male patients with classic FD in the phase 2 study were matched with placebo‐ or agalsidase beta–treated patients from the phase 3 study based on propensity scores at baseline. Changes from baseline in plasma globotriaosylceramide (GL‐3 or Gb3) concentrations were analyzed at approximately 6–36 months.

**Results:**

Venglustat treatment resulted in greater significant reductions in plasma GL‐3 concentrations at 6 months from baseline vs. placebo (mean difference −2.56 μg/mL, *p* < 0.001), and at 24 and 36 months from baseline vs. agalsidase beta (mean difference −1.8 μg/mL, *p* < 0.05 and −2.35 μg/mL, *p* < 0.01, respectively). GL‐3 concentrations continued to decline with venglustat for up to 3 years without plateauing.

**Conclusions:**

Venglustat showed significantly greater reductions in plasma GL‐3 concentrations than placebo after 6 months and agalsidase beta after 24 and 36 months. These findings support the potential of long‐term venglustat treatment to reduce GL‐3 accumulation in patients with classic FD. Further studies are needed to confirm clinical benefit.

## 1 Introduction

Fabry disease (FD, OMIM #301500) is an X‐linked lysosomal disorder resulting from pathogenic variants in the *GLA* gene, which encodes the enzyme α‐galactosidase A (α‐Gal A) (Germain 2010). It is classified into classic or later‐onset forms. Patients with the severe, classic form exhibit low or undetectable α‐Gal A activity and experience early and progressive buildup of glycosphingolipids, particularly globotriaosylceramide (GL‐3 or Gb3), within lysosomes in multiple cell types leading to potentially life‐threatening complications such as strokes, cardiac arrhythmias, cardiomyopathy, and kidney failure (Germain 2010).

In classic patients, clinical manifestation generally begins in childhood and often includes neuropathic pain, impaired sweating, gastrointestinal symptoms, and signs such as *cornea verticillata* and angiokeratomas (Germain 2010). Untreated patients are at high risk of progressive decline in vital organs (kidney, heart, and brain) as they reach adulthood (Germain 2010; Laney et al. 2015; Ortiz et al. 2018; Waldek et al. 2009).

Treatments include enzyme replacement therapy (ERT) with lifelong biweekly intravenous infusions with either recombinant agalsidase beta, agalsidase alfa, or pegunigalsidase alfa. ERTs restore endogenous α‐Gal A activity, thereby clearing accumulated glycosphingolipids (Ortiz et al. 2018; Germain, Arad, et al. 2019; Germain, Elliott, et al. 2019). Although the efficacy and tolerability of ERT for FD are well established, certain gaps remain concerning immunogenicity (Lenders and Brand 2021), optimal dosing, burden of treatment, and optimal time to treatment initiation. An oral pharmacological chaperone, migalastat, is available (Germain et al. 2016) but is suitable only for patients with amenable *GLA* pathogenic variants (Benjamin et al. 2017).

Substrate reduction therapy (SRT) offers an alternative approach for patients with glycosphingolipid metabolism disorders, by inhibiting enzymatic conversion of ceramide to glucosylceramide‐1 (GL‐1) by glucosylceramide synthase (GCS) in the endoplasmic reticulum. GL‐1 is a metabolic precursor of complex glycosphingolipids, including GL‐3 (Platt et al. 2018). Reducing GL‐1 synthesis through SRT has proven safe and effective in patients with non‐neuronopathic, type 1 Gaucher disease (Mistry et al. 2021).

Venglustat, an investigational oral glucosylceramide synthase inhibitor (GCSi), represents a novel approach for treating FD, potentially offering advantages over current treatments. Data from preclinical research (Ashe et al. 2015) and a phase 2 study (Deegan et al. 2023) suggest sustained reduction in plasma GL‐3 and lyso‐GL‐3 over time, consistent with the mechanism of action. However, the biological response to venglustat treatment compared with natural history and ERT in patients with FD is not well established. In this study, we compared the effect of venglustat on reduction in plasma GL‐3 in FD in a single‐arm phase 2 study with matched patients in (a) a placebo arm over 6 months, and (b) an agalsidase beta‐treated arm over 3 years in a historical placebo‐controlled study (Eng et al. 2001).

## 2 Patients and Methods

### 2.1 Ethical Compliance

This study was conducted in accordance with the Declaration of Helsinki and Good Clinical Practice guidelines. The protocols for both the phase 2 venglustat study and the phase 3 agalsidase beta study were reviewed and approved by independent ethics committees or institutional review boards. All participants provided written informed consent prior to enrollment. The analysis presented here is based on historical control and case‐matched comparisons using de‐identified data from previously conducted clinical trials. No new patient recruitment or intervention was involved. Data confidentiality and patient privacy were maintained throughout the analysis in compliance with applicable data protection regulations.

### 2.2 Study Design

ACT13739 was an open‐label, single‐arm, phase 2 26‐week clinical study (NCT02228460) followed by a 130‐week extension study (NCT02489344) that assessed the safety, pharmacodynamics, pharmacokinetics, and exploratory efficacy of 15 mg once daily oral venglustat in treatment‐naïve adult males with classic FD (Deegan et al. 2023).

AGAL‐1‐002‐98 was a double‐blinded phase 3 study wherein patients received either 1 mg/kg of agalsidase beta or placebo every 2 weeks for 20 weeks (Eng et al. 2001). Primary endpoint included change in kidney interstitial capillary GL‐3 and assessment of skin, heart, and plasma GL‐3 compared to baseline. AGAL‐005‐99, an open‐label extension study, treated patients from both arms of AGAL‐1‐002‐98 with 1 mg/kg of agalsidase beta every 2 weeks for an additional 54 months (Germain et al. 2007). Fifty‐eight patients were randomized in AGAL‐1‐002‐98, with 29 patients in each arm, all completing the study. Forty‐four patients completed the open‐label extension study (Germain et al. 2007).

In this study, outcomes were assessed in a historical control matched analysis comparing venglustat‐treated patients from ACT13739 and its 130‐week extension study with placebo‐ and agalsidase beta‐treated patients from AGAL‐1‐002‐98 and its extension study, based on a set of predefined criteria. The primary results of both studies have been published previously (Deegan et al. 2023; Eng et al. 2001; Germain et al. 2007). Similar inclusion criteria and baseline characteristics between the two primary studies justify analyses of venglustat compared to placebo and agalsidase beta on its effect on plasma GL‐3.

Patient matching was performed based on propensity scores to balance patient variables in the venglustat and control (placebo or agalsidase beta) groups for sex, baseline age, plasma GL‐3, urine protein‐to‐creatinine ratio (UPCR), and estimated glomerular filtration rate (eGFR). The AGAL‐1‐002‐98 study included more patients; variable matching was used (1:X) which allowed all available patients to be matched to venglustat patients for additional statistical power.

### 2.3 Measured Outcomes

The endpoint of interest was change in plasma GL‐3 from baseline to up to 36 months measured by liquid chromatography–tandem mass spectrometry in both studies with internal controls to ensure consistency of results over time. The objective endpoint and consistent methodology reduce the risk of bias (Deegan et al. 2023; Eng et al. 2001; Wilcox et al. 2004).

### 2.4 Statistical Analysis

In the matched cohort, paired comparisons were performed to determine the change in baseline plasma GL‐3 by using spaghetti plots over time, overlaid with mean ± SD plots. At each time point, a *t*‐test was used to compare the change from baseline plasma GL‐3 between the two treatments. For the venglustat and placebo comparison, the 6‐month time point (20 weeks of placebo from AGAL‐1‐002‐98 and 26 weeks of venglustat from ACT13739) from baseline was analyzed since placebo data was available only up to 20 weeks. To compare venglustat with agalsidase beta, time points of 6, 12, 24, and 36 months from baseline were analyzed based on availability of data. For 1:X matched analyses, patients were weighted by the inverse of their frequency in the 1:X matched dataset.

For all analyses, a significance level of α = 0.05 was used without any adjustment for multiplicity. All statistical analyses were conducted using SAS statistical‐software‐V.9.2 (SAS Institute, Cary, NC, United States).

## 3 Results

### 3.1 Patient Disposition and Characteristics

Eleven venglustat patients in the ACT13739 study were 1:X matched with placebo and agalsidase beta patients in AGAL‐1‐002‐98 and its extension study, resulting in 19 placebo patients and 28 agalsidase beta patients. All analyzed patients were male and had elevated plasma GL‐3, UPCR < 500 mg/g, and eGFR ≥ 80 mL/min/1.73 m^2^. The baseline characteristics of FD patients treated with venglustat, placebo, and agalsidase beta before and after matching are described in Table 1. Age and baseline plasma GL‐3 were similar after 1:X matching. Specifically, mean age was comparable across the venglustat (26.55 ± 7.61 years), matched placebo (25.65 ± 9.14 years), and matched agalsidase beta (26.03 ± 8.75 years) groups. Mean baseline plasma GL‐3 concentrations were well‐balanced between venglustat (8.86 ± 1.82 μg/mL), matched placebo (10.37 ± 2.21 μg/mL), and matched agalsidase beta (9.71 ± 2.08 μg/mL) groups (upper limit of normal [ULN] 7.03 μg/mL). Individual patient trajectories of plasma GL‐3 in venglustat‐treated patients have been reported in Appendix A of the Supporting Information of the phase 2 study (Deegan et al. 2023).

**TABLE 1:** Baseline characteristics of patients with FD treated with venglustat, placebo, and agalsidase beta.

Matching criteria	Venglustat (*N* = 11)	Placebo		Agalsidase beta^a^	
		Before matching (*N* = 29)	After matching (*N* = 19)	Before matching (*N* = 58)	After matching (*N* = 28)
Age, mean (SD)	26.55 (7.61)	28.78 (11.07)	25.65 (9.14)	30.21 (10.3)	26.03 (8.75)
Plasma GL‐3, μg/mL, mean (SD)	8.86 (1.82)	10.17 (2.59)	10.37 (2.21)	10.05 (2.97)	9.71 (2.08)
UPCR < 500 mg/g, *N* (%)	11 (100)	21 (84)	19 (100)	36 (69)	28 (100)
eGFR ≥ 80 mL/min/1.73 m^2^, *N* (%)	11 (100)	27 (93.1)	19 (100)	55 (95)	28 (100)
Sex: male, *N* (%)	11 (100)	29 (100)	19 (100)	56 (97)	28 (100)

Abbreviations: eGFR, estimated glomerular filtration rate; FD, Fabry disease; GL‐3, globotriaosylceramide; SD, standard deviation; UPCR, urine protein‐to‐creatinine ratio.

^a^ Includes both agalsidase beta/agalsidase beta and placebo/agalsidase beta arms: baseline value was the last measurement before the beginning of agalsidase beta treatment.

### 3.2 Comparison of Venglustat to Placebo

**FIGURE 1:**
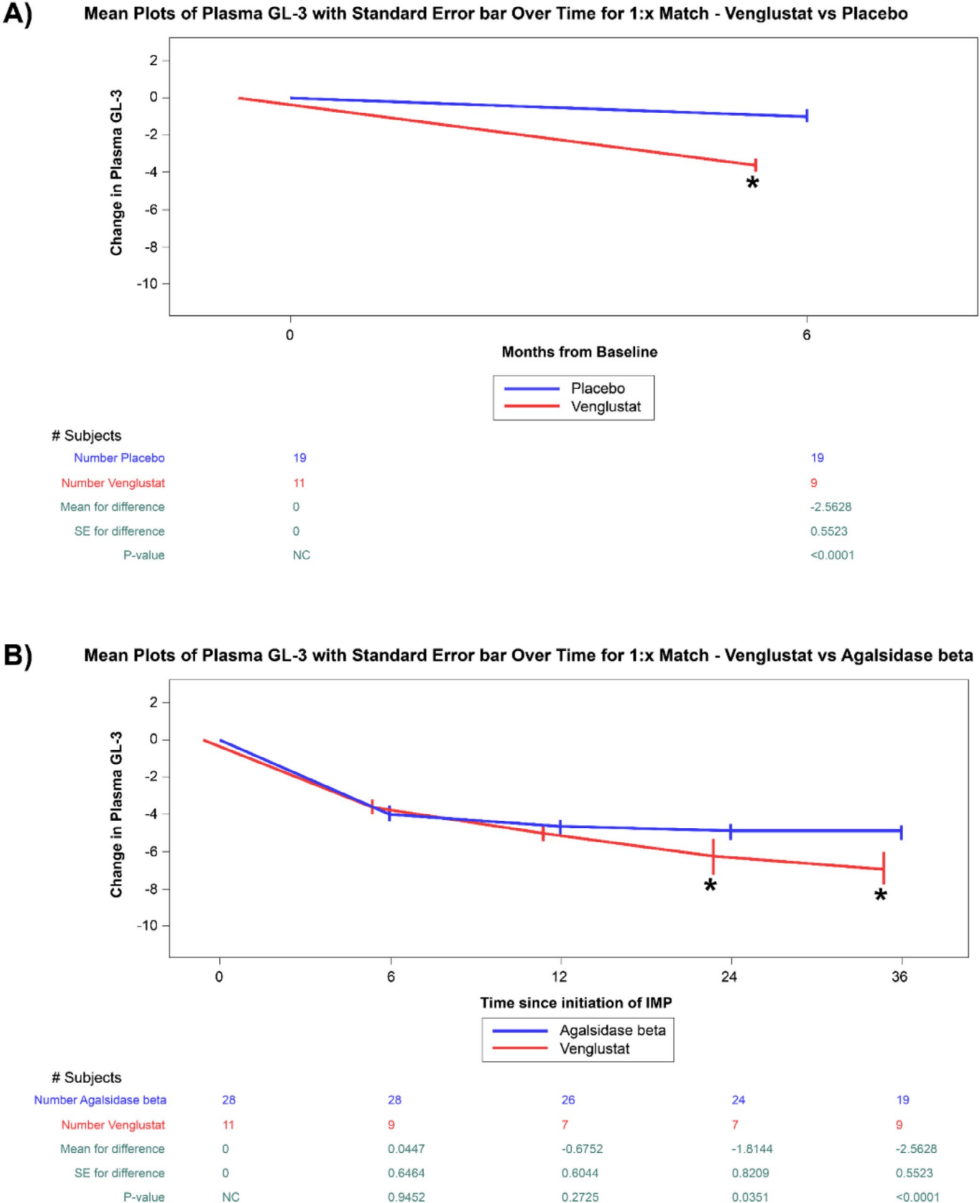
Change in plasma GL‐3 concentrations over time in placebo, venglustat, and agalsidase beta. (A) Plots of changes in plasma GL‐3 from baseline to approximate 6 months in patients with FD treated with venglustat compared to 1:X matched placebo patients (mean ± SE). (B) Plots of changes in plasma GL‐3 concentration from baseline to approximate 6, 12, 24, and 36 months in patients with FD treated with venglustat compared to 1:X matched patients treated with agalsidase beta (mean with standard error). *Indicates *p* < 0.05. FD, Fabry disease; GL‐3, globotriaosylceramide; SE, standard error; vs, versus.

The analysis focused on the change in plasma GL‐3 concentrations from baseline in venglustat‐treated patients after 6 months (26 weeks) of treatment vs. the change from baseline plasma GL‐3 in matched placebo patients after 20 weeks of treatment in their respective studies. The observed mean change in plasma GL‐3 from baseline was −3.62 μg/mL (41.66%) in the venglustat and −1.06 μg/mL (10.22%) in the placebo groups, and the mean difference between the venglustat group at 6 months and the placebo group at 20 weeks was −2.56 μg/mL (*p* < 0.001; Figure 1A).

### 3.3 Comparison of Venglustat to Agalsidase Beta

Reduction in plasma GL‐3 was similar between the two groups at 6 and 12 months (mean difference: 0.04 μg/mL, *p* ≥ 0.05 and −0.60 μg/mL, *p* ≥ 0.05 respectively; Figure 1B), but there was a significantly greater decrease in GL‐3 with venglustat treatment at the time points of 24 and 36 months (mean difference: −1.8 μg/mL, *p* < 0.05 and −2.35 μg/mL, *p* < 0.001 respectively; Figure 1B). In terms of absolute and percentage changes, the observed mean change in plasma GL‐3 from baseline was −6.97 μg/mL (77.50%) in the venglustat group and −4.62 μg/mL (47.58%) in the agalsidase beta group at 36 months. Moreover, plasma GL‐3 values normalized for all venglustat treated patients below the ULN (7.03 μg/mL) by week‐52.

## 4 Discussion

In this study, we analyzed the short‐ and long‐term data from the phase 2 venglustat study combined with the randomized, placebo‐controlled phase 3 agalsidase beta study using a historical case–control method. This post hoc analysis evaluated the potential of venglustat in treating patients with classic FD by comparing the reduction in plasma GL‐3, with both placebo and agalsidase beta treatments. Change in plasma GL‐3 after approximately 6 months of venglustat treatment was compared with matched placebo patients at 20 weeks as a control. Venglustat treatment resulted in a significant reduction in plasma GL‐3 compared to placebo, with a mean difference of −2.56 μg/mL (*p* < 0.001). This confirms the findings of a significantly and progressively decreased plasma GL‐3 from baseline after 6 months of venglustat treatment in the open‐label, single‐arm study with a historical placebo control (Deegan et al. 2023).

To compare the long‐term treatment effects of venglustat with agalsidase beta, we analyzed 11 venglustat‐treated patients against propensity score‐matched male patients with classic FD from the phase 3 and its extension study of agalsidase beta (up to 36 months of treatment). Similar results were observed at months 6 and 12. At 24 and 36 months, venglustat showed a significantly greater reduction in plasma GL‐3 compared to agalsidase beta (*p* < 0.05 and *p* < 0.01 respectively). While these findings suggest that venglustat may have the potential to achieve greater biomarker reduction over the current standard of care direct comparative conclusions should not be drawn in the absence of head‐to‐head phase 3 trial data, even though plasma GL‐3 is a disease‐specific biomarker in FD (Deegan et al. 2023; Eng et al. 2001; Aerts et al. 2008). This finding is also consistent with the mechanism of action of GCSi and shows that the glycosphingolipid burden in FD with venglustat requires long‐term treatment to rebalance the dynamics between synthesis (targeted by venglustat) and degradation of GL‐3 (plausibly through α‐Gal B mediated pathway in the absence of α‐Gal A activity or through conversion to globotriaosylsphingosine and subsequent modification and excretion in urine) (Tomasic et al. 2010; Abe et al. 2000). Interestingly, while receiving ERT plasma GL‐3 plateaued at around 24 months, and plasma GL‐3 progressively decreased up to 36 months while receiving venglustat. Future studies may address whether concentrations continue to decrease thereafter and their impact on drug efficacy and safety.

The reduction in plasma GL‐3 should also be interpreted in the context of the clinical progression of the disease. Encouragingly, in the same population from the phase 2 study, we observed a similar temporal trend in skin GL‐3 reduction (determined via light and electron microscopy) with no significant clinical progression after 3 years of venglustat administration in the young males with classic phenotype (Deegan et al. 2023).

These studies have limitations due to the lack of comparative data based on histological or clinical endpoints, as well as lyso‐GL‐3. While plasma GL‐3 is a disease‐specific biomarker, lyso‐GL‐3 is considered more sensitive and has been shown to correlate more closely with disease activity and severity in FD (Burlina et al. 2023). However, lyso‐GL‐3 data were unavailable for this analysis because the assay was introduced after the AGAL‐1‐002‐98 and AGAL‐005‐99 studies, and the samples from the two studies are not available anymore for reanalysis on lyso‐GL‐3. Patients with significant end organ involvement (eGFR < 60 mL/min/1.73 m^2^ or UPCR ≥ 0.5 mg/g) were excluded in the phase 2 venglustat trial for ethical reasons (risks associated with withholding approved treatments in high risk patients). As a result, baseline eGFR values were within the normal range (mean eGFR: 119.0 and range: 89.0–155.0 mL/min/1.73 m^2^), and no notable changes were observed in either mean or individual patient eGFRs throughout the study period. Similarly, none of the patients exhibited significant proteinuria (> 300 mg/g) at baseline or during the study. Plasma hs‐cTnT concentrations were below the detection limit for all patients at baseline and remained stable at the end of the phase 2 study. Therefore, it is not feasible to compare the effect of venglustat on these clinically meaningful biomarkers with agalsidase beta. In addition, due to the very small sample size, a robust comparison of clinical outcomes was also not feasible.

Regardless, the GL‐3 accumulation in cells leads to damage in vital organs such as the kidneys, heart, and brain, contributing to the clinical manifestations of FD, and plasma GL‐3 concentrations have been used to evaluate severity of the disease and monitor the effectiveness of ERT. Successful treatment typically results in a reduction of both circulating GL‐3 and tissue GL‐3 concentrations, indicating a decrease in lysosomal storage and an improvement in cellular function (Aerts et al. 2008).

The analyses were also limited to classic male patients, and therefore, the results cannot be generalized to the entire FD population. The observed effect in classic male patients suggests that venglustat may have the potential to treat all Fabry variants by reducing glycolipid synthesis and aiding in the degradation of accumulated substrates (Abe et al. 2000). Finally, we demonstrated that venglustat treatment reduced a biomarker GL‐3 in the synthetic and degradative pathway of major glycosphingolipids, confirming target engagement and promising pharmacodynamic effect of venglustat in adult males with classic FD. However, the relevance of the observed response to biomarkers and its correlation with clinical benefit require further characterization once additional data from phase 3 trials, PERIDOT (EFC17045; NCT05206773) and CARAT (EFC16158; NCT05280548), become available.

In conclusion, treatment with venglustat demonstrated a significantly greater reduction in plasma GL‐3 compared to placebo after 6 months, and a significantly greater reduction in plasma GL‐3 compared with agalsidase beta after 24 and 36 months; and contrary to agalsidase beta, did not appear to plateau during follow‐up. These findings support the potential of long‐term venglustat treatment to reduce the GL‐3 accumulation in individuals with FD. The totality of data from the phase 2 study (Deegan et al. 2023) along with this additional historical control case‐matched analysis suggests that venglustat has the potential for the long‐term treatment of FD. Ongoing, larger, randomized, placebo‐ or active‐control, parallel treatment, phase 3 studies will further test the efficacy and safety of venglustat treatment.

## Author Contributions

**Dominique P. Germain:** conceptualization, investigation, supervision, writing – review and editing. **Pronabesh DasMahapatra:** conceptualization, formal analysis, funding acquisitions, investigation, methodology, resources, supervision, writing – review and editing. **Shiguang Liu:** formal analysis, investigation, methodology, resources, supervision, writing – original draft. **Patrick Deegan:** conceptualization, investigation, supervision, writing – review and editing. **Alberto Ortiz:** conceptualization, supervision, writing – review and editing. **Vijay Modur:** conceptualization, funding acquisitions, investigation, methodology, resources, supervision, writing – review and editing. **William R. Wilcox:** conceptualization, investigation, supervision, writing – review and editing. All the authors have critically reviewed the article for important intellectual content.

## Conflicts of Interest

The sponsor had a role in the study design, data analysis, and data interpretation. D.P.G. has received consulting honoraria from Chiesi, Idorsia Pharmaceuticals, Sanofi, and Takeda, and speaker honoraria and travel support from Chiesi, Sanofi, and Takeda and is an investigator in clinical studies for FD sponsored by Chiesi, Idorsia Pharmaceuticals, and Sanofi. P.D.M. and S.L. are employees of Sanofi and may hold stock and/or stock options in that company. P.D. consults with Amicus Therapeutics, Sanofi, and Takeda; has been an investigator in clinical trials sponsored by Amicus Therapeutics, Protalix Biotherapeutics, Sanofi, and Takeda; has received research funding from Sanofi; has received speaker honoraria from Sanofi and Takeda; and has received travel reimbursement from Sanofi. A.O. consults with Sanofi and Chiesi and has speaker honoraria and travel support from Sanofi and Chiesi. V.M. is a former employee of Sanofi and may have held stock and/or stock options in that company. W.R.W. consults with Sanofi, Uniqure, and Relay, and is an investigator in clinical studies for FD sponsored by Amicus Therapeutics, 4D Molecular Therapeutics, Chiesi, Sangamo Therapeutics, and Sanofi. These activities are monitored and are in compliance with the conflicts of interest policies at Emory University School of Medicine.

## Data Availability

Data are available upon request from the corresponding author.
